# Associations between green area in school neighbourhoods and overweight and obesity among Norwegian adolescents

**DOI:** 10.1016/j.pmedr.2017.05.020

**Published:** 2017-06-01

**Authors:** Christine Koteng Wilhelmsen, Katrine Skalleberg, Ruth Kjærsti Raanaas, Håvard Tveite, Geir Aamodt

**Affiliations:** a Department of Public Health Science, Faculty of Landscape and Society, Norwegian University of Life Sciences, Ås, Norway; b Faculty of Sciences and Technology, Norwegian University of Life Sciences, Ås, Norway

**Keywords:** Body mass index, Overweight, Obesity, Geographic information systems, Greenspace, Adolescent, Schools

## Abstract

The aim of the present study was to investigate the relationship between green areas and adolescents' body mass index (BMI). This is based on the notion that nature environment is known to have beneficial effects on human health, and that some of the explanation for this is that green areas are especially motivating or suitable as arenas for physical activity. We included 10,527 participants from the Norwegian Youth Study, which was conducted between 2001 and 2004. The participants reported body weight, height, and important potential confounding variables about lifestyle, family situation, and neighbourhood characteristics. Green area was assessed from land cover maps and we calculated the percentage of green areas within 1 km and 5 km buffers around the adolescents' schools. We found that the percentage of overweight and obese adolescents increased significantly when the percentage of green areas in the participants' surrounding increased (*p* < 0.001 for both outcomes and buffer sizes). The same results were found in logistic regression models where we adjusted for a large set of variables. As an example, the odds for being overweight was 1.38 times higher (95% CI: 1.02–1.85) for participants living in the most green surroundings compared to participants living in the least green surroundings (1 km buffer). Norwegian green areas are typically farmland, woods, and mountains, and we speculate if these areas are less accessible and attractive for adolescents, who might need more facilitated green areas for sport and physical activity.

## Highlights

• Prevalence of overweight among Norwegian adolescents varies dependent on greenness.
• The prevalence is highest in most green areas and lowest in less green areas.
• Physical activity and active transportation are independent explanatory factors.
• Health promoting activities should be understood better in rural areas in Norway.

## 1 Introduction

Overweight and obesity among children and adolescents are global problems. According to the World Health Organization (WHO), 42 million children under 5 years were overweight or obese in 2015 (WHO, 2016). Overweight and obesity among children and adolescents can lead to troubles in breathing, increased risk of fractures, hypertension, insulin resistance, psychological effects, and increased risk of overweight and obesity in adulthood (WHO, 2003). Among adults, overweight and obesity can lead to cardiovascular diseases, musculoskeletal disorders, cancer, and diabetes type 2 (WHO, 2008). Prevention of overweight and obesity should start as early as possible, and if we start in childhood or adolescence, serious physical, social, and psychological consequences can be prevented (WHO, 2014).

The mechanisms leading to overweight and obesity are complex, and they are linked to both lifestyle, the environment, and genetics. Overweight and obesity are caused by energy intake exceeding the energy expenditure, i.e. an imbalance between food intake and how much energy they use (Han et al., 2010). Some genes increase the risk of overweight and obesity (Mutch and Clement, 2006), but the large increase in overweight and obesity during the past 30 years cannot be explained by genetics alone. The causes are more likely connected to the environment and lifestyle factors (Ebbeling et al., 2002).

The environment affects human health, and studies have found significant associations between green surroundings and physical and mental health (Hartig et al., 2014, James et al., 2015). Lachowycz and Jones suggested three groups of explanations, which in different ways include the physical environment's ability to create changes in individuals' health behaviour (Lachowycz and Jones, 2013); nature's capabilities for restitution and aesthetic satisfaction, social interactions within greenspace, and possibilities for health promoting physical activities.

Several studies have investigated the relationships between green areas and overweight and obesity. Most studies have examined adults (Astell-Burt et al., 2014, Bjork et al., 2008, Coombes et al., 2010, Cummins and Fagg, 2012, Michimi and Wimberly, 2012, Mowafi et al., 2012, Nielsen and Hansen, 2007, Pereira et al., 2013, Prince et al., 2012, Prince et al., 2011, Richardson et al., 2013, Rundle et al., 2013), but some have examined children and adolescents (Bell et al., 2008, Burgoine et al., 2015, Dadvand et al., 2014, Liu et al., 2007, Lovasi et al., 2013, Potestio et al., 2009, Potwarka et al., 2008, Wall et al., 2012, Wolch et al., 2011, Groholt et al., 2008). Different definitions of green areas have been used in these studies, but the majority have used distance to parks (Dadvand et al., 2014, Liu et al., 2007, Potestio et al., 2009, Potwarka et al., 2008, Wall et al., 2012, Wolch et al., 2011). Several studies focus on the interaction between green area, physical activity, and bodyweight such as Potestio and co-workers (Potestio et al., 2009). Most of the studies were conducted in urban areas, but some were also conducted in rural areas (Bell et al., 2008, Groholt et al., 2008, Sjoberg et al., 2011).

The aim of this study was to investigate the association between green areas surrounding adolescents' schools and overweight and obesity among Norwegian adolescents. We also wanted to investigate how specific variables, which could initiate a difference in health behaviour, modify or mediate the relationship between greenspace and health. These variables were adolescents' use of nature, physical activity level, and mode of transportation to school.

## 2 Methods and material

**Fig. 1:**
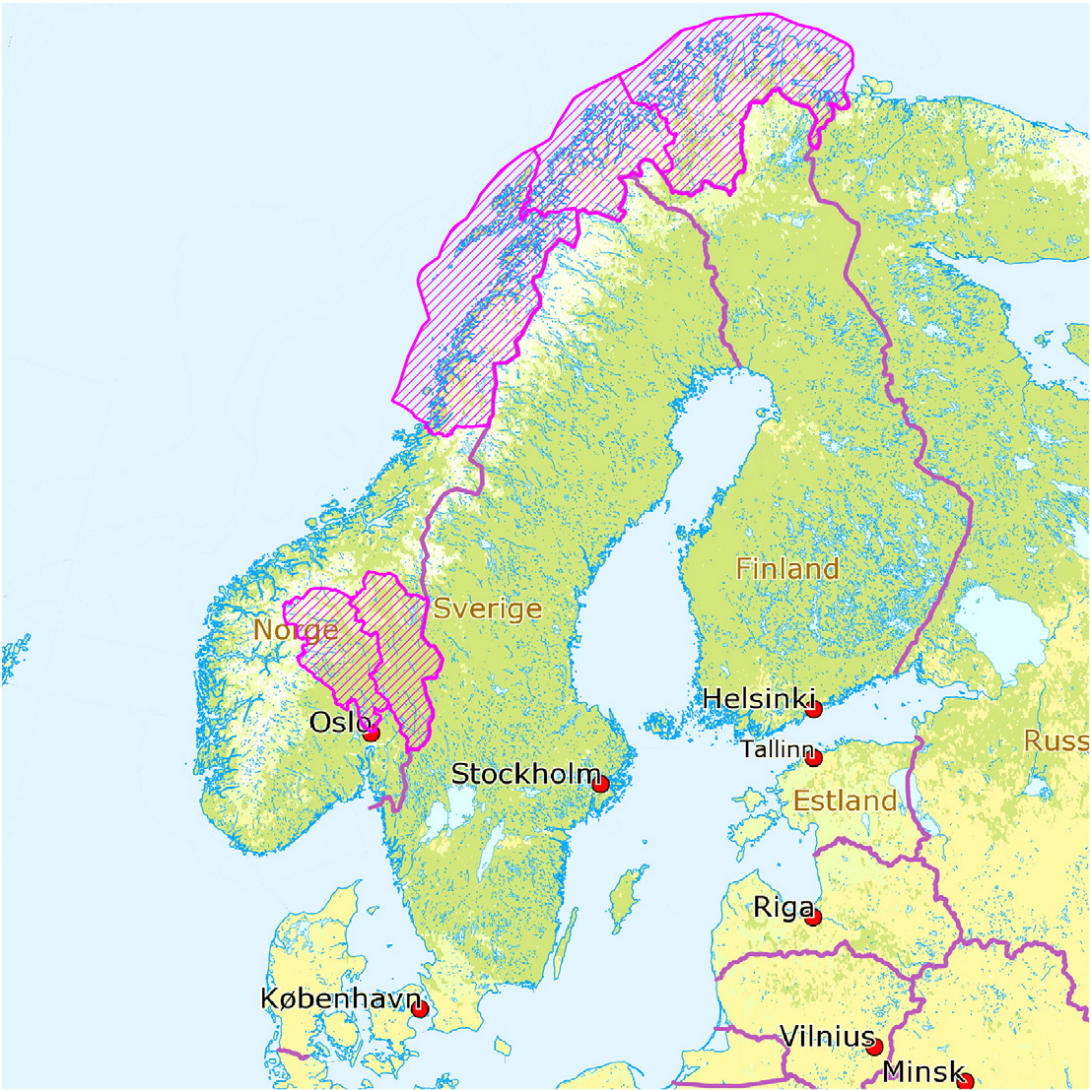
Adolescents in six counties participated in the Norwegian Youth Study 2001–2004.

This cross-sectional study was based on data from the *Norwegian Youth Studies* conducted by University of Tromsø, University of Oslo, and The Norwegian Institute of Public Health between 2001 and 2004. The school-based survey collected information on health related issues among Norwegian adolescents, in addition to diet, smoking habits, life events, physical activity and sport, family relations, and welfare and living conditions in six of 19 counties in Norway (Fig. 1). All 10th graders (predominantly 15 or 16 years old) were invited to participate. A total of 15,966 adolescents from 356 schools answered the questionnaires:

7342 in Oslo, 1939 in Hedmark, 1877 in Oppland, 2657 in Nordland, 1514 in Troms, and 637 in Finnmark. The response rate was 86.4% (Groholt et al., 2008). The size of the complete-case dataset with relevant variables was 10,527. All data from The Norwegian Youth Studies are self-reported.

### 2.1 Geographical variables

**Fig. 2:**
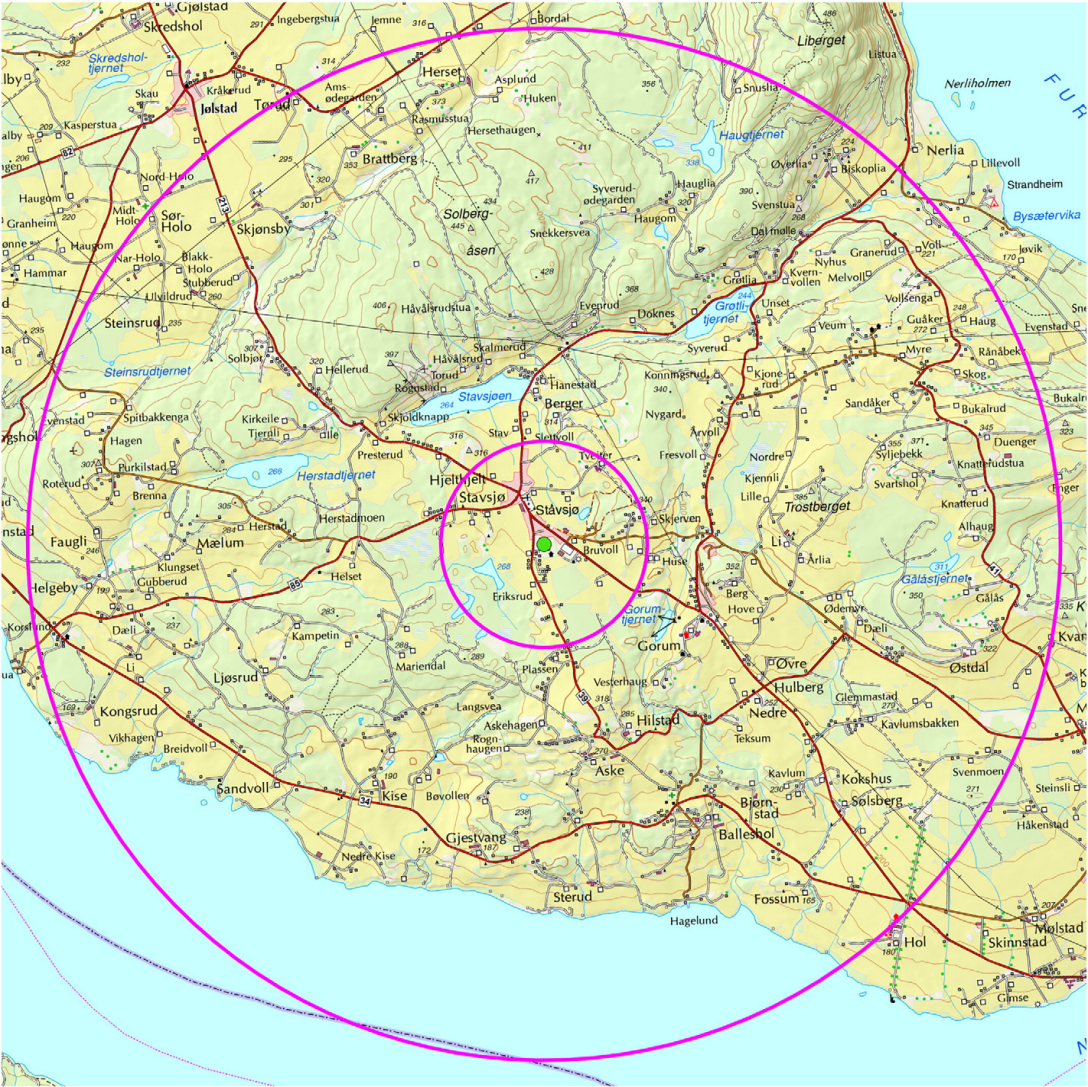
An example of two buffers with radii 1 km and 5 km surrounding a typical school. The Norwegian Youth Study, 2001–2004.

We used a Geographical Information System (GIS) to determine the percentage of green areas around schools. First, all the schools were geocoded. Next, we produced buffers with radii of 1000 m and 5000 m around each school, and then we computed the amount of green areas within each buffer (Fig. 2). Green area was retrieved from land cover maps downloaded from the Norwegian Mapping Authority's website. We selected the following attributes to represent green areas: park, forest, open area, sports arena, alpine hill, cropland, river and stream, fresh water dry fall, golf course, graveyard, ocean surface, lake, and marsh. We also produced green area variables without the “open area” attribute. The green area variable was included in the analyses as both continuous and categorical variables. For the latter, we divided the variables into five categories (quintiles), representing five degrees of greenness, ranging from 1 (least green) to 5 (most green). We also created a dichotomous variable based on the categorical variable of green area in a buffer of 1 km. “Least greenness” included the two quintiles with the least green areas and “Most greenness” included the three quintiles with the most green areas.

We included the climatic variables precipitation and temperature, as well as altitude. Climatic variables were downloaded from www.eklima.no. We used ArcGIS, version 10.2.2 (ESRI, Redlands, CA) to produce the greenness variables.

### 2.2 Outcome variables

The outcome variable Body Mass Index (BMI) was calculated by dividing self-reported weight (in kilograms) by height (in meters) squared. To define underweight, normal weight, overweight and obesity, we used Cole and colleagues' age- and sex-specific BMI-classification (Cole et al., 2000). In the present study, 15.5 years was used as BMI reference as the study population was predominantly 15 or 16 years of age, with a mean age of 15.36 years. Cut offs for underweight was < 17.26 kg/m^2^ for boys and < 17.69 kg/m^2^ for girls. Normal weight was 17.27–23.59 kg/m^2^ for boys and 17.70–24.16 kg/m^2^ for girls. Overweight was 23.60–28.59 kg/m^2^ for boys and 24.17–29.28 kg/m^2^ for girls. Obesity was above 28.60 kg/m^2^ for boys and above 29.29 kg/m^2^ for girls (Cole et al., 2000). We created dichotomous variables for overweight and obesity, based on the criteria defined above. The variable for overweight included obese participants. We included participants with height between 140 and 200 cm, based on growth scales for the age group (Juliusson et al., 2009). We restricted BMI to 14–60 kg/m^2^.

### 2.3 Potential mediators and confounders

Physical activity was measured as a variable grouped into two categories, which follow the national recommendations for physical activity for adolescents: “less than 7 h a week” or “more or equal to 7 h a week”. We included two questions about transportation to school: “How do you get to school in the summer season?” We grouped the variables into two categories accommodating passive transportation (car/bus/train or similar) or active transportation (moped/bicycle/walking). We assessed the adolescents' use of nature based on the questions: “Do you use the nature (woods and fields) for walks/trips more than once a month?” All potential mediators were also examined as possible effect modifiers, which is described below.

A set of confounders was also included. The adolescents' ethnicity was categorized into three groups based on the parents' country of birth: “Both parents Norwegian”, “One parent foreign”, “Both parents foreign”. We produced a diet-related variable summing up a set of frequency-based items of healthy food. The variable was later categorized into a dichotomous variable with the levels low and high score. Questions regarding smoking habits and use of non-smoking nicotine were categorical variables with four categories and later regrouped into the categories: “Not smoking”, “Current smoking”. To investigate the socio-economic situation in the family in this study, we included three variables: “How is the financial situation in your family?”, “Is your father/mother currently employed?”, and “Whom do you currently live with?” The latter question had the following categories: “Mother and father”, “Only mother”, “Only father”, “About as much with mother and father”, “Mother or father and new partner (co-habitant) or spouse”, “Foster parents”, “Other”. “How is your parents' domestic situation?” had the categories “Married/living together”, “Not married”, Divorced/separated”, “One or both are dead”, “Other”. We regrouped these categories into living in a family with more than one parent and living in a family with only one parent. The variables accommodating social support from friends and family were computed from several statements. “Social support from family” is the sum of responses from five statements such as: “I feel close to my family”, “I am being taken seriously by my family”, and “I can count on my family when I need help”. The variable “Social support from friends” is the sum of responses from four statements such as: “I feel very close to my friends”, and “I can count on my friends when I need help”. The variables were categorized into “Good” or “Poor”.

Finally, we included county and moving history, where moving history included four categories: “No”, “Yes, once”, “Yes, 2–4 times”, “Yes, 5 times or more”.

### 2.4 Statistical analysis

We used Pearson's Chi-square to investigate dependencies between categorical variables such as overweight and groups of green area, and *t*-tests to investigate if groups of continuous variables were distributed differently. Logistic regression analysis was used to investigate the association between green area and overweight/obesity. We fitted two regression models. In the first model, we adjusted for age, gender, and ethnicity. In the second model, we also adjusted for physical activity, transportation mode, use of nature, questions about family situation, diet, smoking habits, county, moving history, and climatic variables (precipitation, altitude, and temperature). In separate models, we included green area as a continuous variable and we reported the resulting regression slopes (including 95% confidence intervals).

To study effect-modification, we included an interaction term between the green area variable and key variables that we were interested in: use of nature, physical activity, and mode of transportation to school.

*p*-values < 0.05 were considered significant and all analyses were performed using the free software R version 3.2.2.

### 2.5 Ethics

This study has been approved by the Regional Committee for Medical and Health Research Ethics (2014/1692). All the parents gave their informed written consent for the adolescents to participate.

## 3 Results

In Table 1, we list characteristics of the participants dependent on the two categories of amount of green area. Many variables were distributed differently dependent on which category of greenness the participants belonged to - least green or most green. Typically, adolescents living in the most green areas were less physically active (*p* = 0.003), used passive transportation more often (*p* < 0.001), used nature more frequently (*p* < 0.001), and used non-smoking nicotine more often (*p* < 0.001) compared to those living in the least green areas. The adolescents' perception of the family economy was more often poor (*p* < 0.001), they lived more frequently with two parents rather than one parent (*p* = 0.036), and their father and mother were less often employed (*p* < 0.001). Participants living in the greenest areas scored lower on the healthy diet variable (*p* < 0.001) than their peers living in least green areas.

**Table 1:** Demographic variables of Norwegian adolescents 2001–2004, based on the Norwegian Youth Study (*n* = 10,527). *P*-values are results from chi-square tests.

		Least greenness	Most greenness	*P*-value
Age	14–15 years	5268 (65.4%)	1585 (64.2%)	0.504
	16–17 years	2791 (34.6%)	883 (35.8%)	
Gender	Man	4063 (50.4%)	1261 (51.1%)	0.919
	Woman	3996 (49.6%)	1207 (48.9%)	
Parents country of birth	Both Norway	6325 (78.5%)	2272 (92.1%)	< 0.001
	One Norway	738 (9.2%)	111 (4.5%)	
	None Norway	996 (12.4%)	85 (3.4%)	
Physical activity	< 7 h per week	4650 (57.7%)	1493 (60.5%)	0.003
	≥ 7 h per week	3409 (42.3%)	975 (39.5%)	
Transport, summer	Bus/car	2647 (32.8%)	1535 (62.2%)	< 0.001
	Walking/bicycle	5412 (67.2%)	933 (37.8%)	
Use of nature	Seldom	5780 (71.2%)	1567 (63.5%)	< 0.001
	Frequent	2279 (28.3%)	901 (36.5%)	
Current smoking	No	5806 (72%)	1724 (69.9%)	0.065
	Yes	2253 (28%)	744 (30.1%)	
Non-nicotine tobacco habits	No	7140 (88.6%)	2128 (86.2%)	0.002
	Yes	919 (11.4%)	340 (13.8%)	
Parents	Married/cohabitant	5522 (68.5%)	1683 (68.2%)	0.817
	Single	2537 (31.5%)	785 (31.8%)	
Perceived family economy	Poor	2700 (33.5%)	1044 (42.3%)	< 0.001
	Good	5359 (66.5%)	1424 (57.7%)	
Moving history last five years	None or one time	7320 (90.8%)	2268 (91.9%)	0.172
	Two or more times	739 (9.2%)	200 (8.1%)	
With whom do you live	Parents. new family, foster parents	6325 (78.5%)	1982 (80.3%)	0.036
	One parent	1734 (21.5%)	486 (19.7%)	
Employment. father	Employed	6671 (82.8%)	1976 (80.1%)	0.001
	Unemployed	1388 (17.2%)	492 (19.9%)	
Employment, mother	Employed	4872 (60.5%)	1379 (55.9%)	< 0.001
	Unemployed	3187 (39.5%)	1089 (44.1%)	
Healthy diet	Low score	4136 (51.4%)	1395 (56.5%)	< 0.001
	High score	3920 (48.6%)	1073 (43.5%)	
Support from family	Good	4788 (59.4%)	1353 (54.8%)	0.001
	Poor	3271 (40.6%)	1115 (45.2%)	
Support from friends	Good	4914 (61%)	1489 (60.3%)	0.583
	Poor	3145 (39%)	979 (39.7%)	
Temperature, centigrade annual mean	Mean, (SD)	4.6 (1.5)	3.2 (1.5)	< 0.001
Precipitation (mm per year)	Mean, (SD)	60.0 (124.7)	151.7 (124.7)	< 0.001
Altitude (m)	Mean, (SD)	802.7 (255.9)	856.3 (255.9)	< 0.001
Overweight	Normal weight	7090 (88%)	2036 (82.5%)	< 0.001
	Overweight	969 (12%)	432 (17.5%)	
Obese	Not-obese	7933 (98.4%)	2392 (96.9%)	< 0.001
	Obese	126 (1.6%)	76 (3.1%)	

In Table 2, we show that the percentage of overweight and obese adolescents increased significantly with increasing percentage of green area within the buffers. The proportion of overweight participants changed steadily as a function of green area within a 1 km buffer from 11.1% (least green), to 13.6%, 16.5%, 19.1%, and to 19.0% (most green). We observed parallel results for the 5 km buffer and for obese adolescents.

**Table 2:** Association between greenness, overweight, and obesity, based on the Norwegian Youth Study, 2001–2004 (*n* = 10,527).

	Buffer length	All	Below 20%	20–40%	40–60%	60–80%	Above 80%	*p*-value
Overweight	1 km	13.3%	11.1%	13.6%	16.5%	19.1%	19%	< 0.001
Overweight	5 km	13.3%	10.2%	15.6%	16.1%	19.1%	21.2%	< 0.001
Obesity	1 km	1.9%	1.2%	2.2%	3.1%	3.1%	3.4%	< 0.001
Obesity	5 km	1.9%	1.2%	2.6%	2.6%	3.3%	3.2%	< 0.001

Table 3 shows the results from the logistical regression models where we adjusted for different sets of variables. In the upper part of the table, we included gender, age, and parents' country of birth; in the lower part, we included a larger set of variables. The results show that the odds for both overweight and obesity increased with increasing percentage of green area. The results are parallel for both buffer sizes and both sets of confounding variables. The fully adjusted odds ratio for overweight was 1.38 (95% CI: 1.02–1.85) times larger in the greenest areas compared to the least green areas within the 1 km buffer. For overweight within the 5 km buffer the parallel result was 2.01 (95% CI: 1.44–2.81). The odds ratios for obesity in the greenest area compared to the least green area were 1.56 (95% CI: 0.79–3.05) in the 1 km buffer and 2.47 (95% CI: 1.10–5.56) in the 5 km buffer. In the right part of the table, we show the results where we included green area as a continuous variable. The linear trend was statistically significant for both combinations of outcomes and buffer sizes.

**Table 3:** Results from logistic regression between green areas (1 km and 5 km buffers) and outcome variables (overweight and obesity) based on the Norwegian Youth Study, 2001–2004 (*n* = 10,527).

	Buffer size	Least greenness	2	3	4	Most greenness	Slope (96% CI)
*Unadjusted^1^*							
Overweight	1 km	1 (ref)	1.27 (1.11–1.46)	1.60 (1.35–1.88)	1.86 (1.48–2.32)	1.87 (1.42–2.47)	0.01 (0.01–0.01)
Overweight	5 km	1 (ref)	1.68 (1.45–1.93)	1.72 (1.45–2.04)	2.13 (1.71–2.66)	2.41 (1.83–3.17)	0.02 (0.02–0.02)
Obesity	1 km	1 (ref)	1.55 (1.09–2.21)	2.36 (1.59–3.48)	2.66 (1.59–4.45)	2.62 (1.4–4.92)	0.02 (0.02–0.03)
Obesity	5 km	1 (ref)	2.54 (1.77–3.64)	2.54 (1.66–3.89)	3.21 (1.92–5.38)	3.09 (1.59–6.01)	0.04 (0.04–0.05)

*Adjusted^2^*							
Overweight	1 km	1 (ref)	1.14 (0.99–1.32)	1.35 (1.12–1.62)	1.38 (1.09–1.76)	1.38 (1.02–1.85)	0.01 (0.01–0.01)
Overweight	5 km	1 (ref)	1.55 (1.25–1.94)	1.67 (1.3–2.14)	1.91 (1.44–2.54)	2.01 (1.44–2.81)	0.02 (0.02–0.02)
Obesity	1 km	1 (ref)	1.35 (0.93–1.95)	1.78 (1.15–2.76)	1.65 (0.95–2.88)	1.56 (0.79–3.05)	0.01 (0.01–0.02)
Obesity	5 km	1 (ref)	2.59 (1.47–4.56)	2.55 (1.37–4.76)	2.68 (1.37–5.24)	2.47 (1.1–5.56)	0.04 (0.04–0.05)

In the “unadjusted” model, we adjusted for age, gender, and ethnicity. In the adjusted model, we also adjusted for physical activity, transportation mode, use of nature, social support from friends and family, family situation, diet, smoking habits, county, moving history, and climatic variables (precipitation, altitude, and temperature).

We report the associations between the variables associated with the explanatory mechanisms and the outcome variables in Table 4. Physical activity and active transportation during the summertime decreased the odds for both overweight and obesity. We did not find parallel results for use of nature. Finally, we included interaction terms between green area and the variables of interest. We did not find any statistically significant effect-modifications (results not shown).

**Table 4:** Results from adjusted regression models. The table shows odds ratios including 95% confidence intervals for variables suggested as mediators based on the Norwegian Youth Study, 2001–2004 (*n* = 10,527).

		Overweight		Obesity	
		1 km	5 km	1 km	5 km
Physical activity	< 7 h per week	1 (ref)	1 (ref)	1 (ref)	1 (ref)
	> 7 h per week	0.78 (0.69–0.88)***	0.78 (0.69–0.88)***	0.51 (0.37–0.71)***	0.51 (0.37–0.71)***
Transport. summer	Bus. Car	1 (ref)	1 (ref)	1 (ref)	1 (ref)
	Walking. bicycling	0.8 (0.71–0.91)***	0.82 (0.73–0.93)**	0.61 (0.45–0.83)**	0.63 (0.46–0.85)**
Use of nature	Seldom	1 (ref)	1 (ref)	1 (ref)	1 (ref)
	Often	0.88 (0.77–1.00)	0.87 (0.76–1.00)*	0.88 (0.63–1.22)	0.88 (0.63–1.22)

*p* < 0.001 (***). *p* < 0.01 (**); *p* < 0.05 (*).

We analysed the associations between greenness and BMI, where we used the variable accommodating green area without the “open area” attribute. This produced similar results (results not shown).

## 4 Discussion

In this study, we found that high percentage of green areas in the school neighbourhood was associated with increased likelihood of being overweight or obese among our study population. We found the same results for both 1 km and 5 km buffers; however, the associations were strongest for the larger buffer zone. We included a set of potential confounding/mediating variables, but they only slightly attenuated the effect sizes. Physical activity and active transportation showed independent significant associations with overweight and obesity. We found no evidence of interaction between green area and use of nature.

Previous research on the relationship between green areas and adolescents' weight has found protective effects or no significant associations, contrary to the results in our study. The assessment and measurement of green areas differ, which is also the case for the set of confounders included in the studies. Liu and co-workers assessed greenness based on remote sensing (satellite data) and within 2 km buffers centred around the participants' homes (Liu et al., 2007). They found that for children residing in densely populated regions, increased percentage of vegetation was related to decreased risk for overweight. Potwarka et al. examined number of parks, distance to parks and percentage of parks within 1 km buffers and their influence on weight in children and adolescents (Potwarka et al., 2008). They found no associations between body weight and the buffer variables; however, children with a park playground within 1 km from their home were five times more likely to have a healthy weight compared to those without a playground. A similar study investigating associations between patterns of obesogenic neighbourhood features and adolescents' weight status, found a protecting role of recreational areas (Wall et al., 2012). In a longitudinal study from 12 communities in Los Angeles, Wolch and colleagues found that proximity to parks within 500 m and number of public recreation programs offered within a 10 km buffer from their homes reduced the risk of overweight and obesity among adolescents over a period of eight years (Wolch et al., 2011). In another longitudinal study based on remote sensing data, Bell and co-authors examined two-year changes in BMI in children and adolescents and amount of green area in the neighbourhood. Higher percentage of green area was associated with lower BMI in the second year, and green area was also associated with lower likelihood of increased BMI scores over two years (Bell et al., 2008).

The prevalence of obesity varies and this could partly explain the differences between the studies. The prevalence of overweight among children and adolescents in Scandinavian countries were about 13–14% (Sjoberg et al., 2011), while the same figures in Los Angeles was 32% (Wolch et al., 2011). Pereira observed that 25% of young adults (15–25 years) in Perth, Western Australia, were overweight (Pereira et al., 2013).

Lachowycz and Jones (Lachowycz and Jones, 2013) have proposed a theoretical framework for better understanding the relationships between greenspace and health. One mechanism is the ability of the greenspace to enhance mental restitution. In our study, adolescents living in the greenest areas did use nature more often than adolescents living in less green areas. However, results from the fully adjusted models showed that use of nature was not associated with the outcome variables and use of nature is therefore not a mediating variable. Secondly, greenspace may be suitable or motivating as arenas for different kinds of physical activities. In this study, physical activity and transportation mode to school during the summer season were both significantly associated with green area. These variables were still significant in the fully adjusted models; however, the effect size between green area and the outcome variables changed insignificantly. We therefore suggest that these variables work as independent factors more than mediators or confounders. It is important to note that the variables we have included to mimic the explanatory mechanisms are only proxies. We were therefore not able to test the theoretical framework proposed by Lachowycz and Jones (Lachowycz and Jones, 2013).

Interestingly, associations between green areas and obesity were more pronounced in studies where one had used distance to parks to assess greenness rather than satellite data. In our study, we use a broader definition of green areas including any green attributes from land-use maps, which in many ways is closer to the assessment of greenness using satellites data and remote sensing. Parks and playgrounds invite individuals to healthy behaviour more than croplands, forests, lakes, and marshes. This could explain why we did not observe any protecting effects of green areas, but it does not explain the positive association we observed.

In a meta-analysis, Johnson and Johnson compared obesity among children and adolescents in rural and urban areas in the United States. The odds for childhood and adolescent obesity was significantly 26% higher for individuals in rural areas than in urban areas (Johnson and Johnson, 2015). Sjöberg found the same association in a Swedish study (Sjoberg et al., 2011); however, this association was confounded by area level education, which we were not able to include in our study. It is likely that our greenest areas correspond to rural areas and less green areas as urban areas. In our study and Johnson and Johnson's meta-analysis, physical activity, active transportation, or diet did not mediate the associations. However, types of nature and access to different facilities differ between urban and rural areas and could explain our findings.

The strength of this study is the large sample of adolescents from six counties in different parts of Norway and the rich set of variables in the questionnaire. All adolescents in 10th grade were invited to join, and the study had a relatively high response rate. We included several different geographical regions in this study and the variables assessing green areas were objectively measured.

Among the limitations of this study is the fact that this is a cross-sectional study, which means we cannot say anything about the causal relationship between the exposure and the outcome variables. The response rate differed between the counties (Groholt et al., 2008), which could cause selection bias. Weight and height, along with the other variables in the youth study, are self-reported. This could result in an information bias. However, research shows that self-reported height and weight in adolescents are accurate for those over 14 years old (Himes and Faricy, 2001). We have included many possible confounding factors in this study, but some confounding factors are not sufficiently accommodated, such as diet. It is not possible to say anything about caloric intake, nor is it possible to assess caloric expenditure. The variable on physical activity may not reflect general activity during the day. Sports and exercise may not reflect how active the adolescents really were. Sedentary time or screen time is not included as a confounder. Parents' socio-economic status and area-level education were not sufficiently included. We included parents' working status as well as perceived family finance, but these variables do not include information about what kind of job or education the parents have. We have not included factors like traffic density, inclination of the green area, or safety, which reflect accessibility of the green areas. Finally, there is a time gap between the youth studies and the maps including the green attributes, which could also produce information bias.

## 5 Conclusion

In this study, we found that increased amount of green areas within school environments increased the odds of overweight and obesity in adolescents. The associations were only slightly attenuated when adjusting for a large set of confounding/mediating variables. Norwegian green areas differ between predominantly cropland, forests, and mountains in rural parts of the country and more facilitated areas in urban areas. It is possible that these areas are less accessible or also less attractive to adolescents, who may prefer more facilitated green areas. This can lead to less physical activity among adolescents in rural areas, which in turn can lead to more overweight and obesity.

The relationship between the nature environment and overweight and obesity seems to be complex, and further studies in the area need to differentiate between green areas in urban and rural areas. For green areas to encourage increased physical activity among adolescents, we need a clear understanding of how the green areas can be facilitated to achieve this.

## Conflict of interests

The authors declare they have no conflicts of interest.
